# Single-shot AF ablation, Leadless pacemakers and LAAO are EP, not IC procedures

**DOI:** 10.1016/j.ipej.2026.03.009

**Published:** 2026-03-05

**Authors:** Tolga Aksu, Mina K. Chung

**Affiliations:** a Department of Cardiology, İstanbul Aydin University, MLPCARE, Medical Park Florya Hospital, Istanbul, Turkey; b Heart, Vascular, and Thoracic Institute, and Cleveland Clinic Research, Cleveland Clinic, Cleveland Clinic Lerner College of Medicine of Case Western Reserve University, Cleveland, OH, USA

## 1 Why this clarion call—why now?

Cardiac electrophysiology (EP) is undergoing a rapid cycle of innovation: single-shot atrial fibrillation (AF) ablation technologies, leadless pacing, and left atrial appendage occlusion (LAAO) are evolving from niche offerings into mainstream therapies. Paradoxically, as these procedures become more “streamlined,” a narrative emerges that they are increasingly “generic catheter skills”—and thus easily transferable to any interventional cardiologist. This editorial argues for a more disciplined framing: these are fundamentally EP procedures, rooted in arrhythmia pathophysiology, electrical diagnosis, procedural electrophysiologic endpoints, and longitudinal rhythm-centric care.

To be clear, the goal is not territorialism. The goal is patient safety, outcome integrity, workforce strategy, and sustainable quality. The question is not who is “allowed” to do what. The question is: what training, governance, endpoints, and follow-up infrastructure are required so that single-shot AF ablation, leadless pacing, and LAAO deliver what the evidence promises—consistently, across real-world systems?

## 2 First principles: what defines an “EP procedure”?

Procedures should be classified not by the shape of the catheter or which lab hosts the case, but by:

Core decision-making domain (electrical vs purely structural/hemodynamic).

Procedural endpoints (electrophysiologic and rhythm outcomes vs anatomic-only).

Complication profile and rescue pathways (arrhythmia-specific complications, device interactions, rhythm emergencies).

Required longitudinal care model (rhythm monitoring, antiarrhythmic management, device programming, anticoagulation strategy, redo strategy).

By these criteria, single-shot AF ablation, leadless pacemakers, and LAAO sit squarely within EP. They demand not only catheter dexterity but electrical interpretation, mapping literacy, device behavior comprehension, and rhythm-focused longitudinal governance.

## 3 Single-shot AF ablation: “Simpler tools” do not simplify AF

Single-shot ablation (cryo-balloon, pulsed-field ablation [PFA], or other single-shot platforms) can compress procedural steps and shorten learning curves for certain mechanical maneuvers [[1], [2], [3]]. However, AF is not a “pulmonary vein plumbing problem.” It is a dynamic arrhythmia syndrome: triggers, substrate, autonomic influences, atrial cardiomyopathy, and comorbidity-driven progression [[4], [5], [6]].

### 3.1 Why EP ownership matters

a) Patient selection is electrophysiological medicine

The difference between durable success and recurrent AF often begins before the sheath enters the septum: phenotype (paroxysmal vs persistent), atrial size and fibrosis surrogates, sleep apnea burden, obesity, alcohol, thyroid disease, autonomic tone, prior flutter circuits, and the likelihood of non-PV triggers [4,[6], [7], [8], [9]]. These are not “nice-to-have” considerations; they determine whether single-shot pulmonary vein isolation (PVI) alone is adequate or insufficient.

b) Endpoints are not purely anatomic

Even in single-shot workflows, EP endpoints matter: entrance/exit block (where applicable), waiting periods, adenosine testing philosophy, trigger provocation, management of reconnection risk, interpretation of far-field signals, ablation of accompanying arrhythmias. Without an EP mindset, “PVI performed” can quietly degrade into “PVI assumed.”

c) Complications and rescue are rhythm-centric

Tamponade and vascular complications are shared procedural risks; but AF ablation carries uniquely EP-heavy rescue pathways: management of atrial tachycardias, iatrogenic flutters, sinus node dysfunction unmasking, atrioesophageal injury vigilance protocols, phrenic nerve monitoring, post-ablation bradyarrhythmias, and the nuanced anticoagulation choreography.

d) Longitudinal outcomes are EP-defined

Success is not only “no AF on discharge.” It is freedom from symptomatic arrhythmia, reduced AF burden, minimized hospitalizations, rational antiarrhythmic use, and stroke prevention strategy over time. That care model is the EP clinic and device/monitoring ecosystem.

The real risk: de-skilling the cognitive layer.

Single-shot platforms tempt systems into treating AF ablation as a volume commodity: “If it's fast, anyone can do it.” That premise risks cognitive de-skilling: fewer operators maintain high-level competence in mapping, redo strategy, atypical flutter diagnosis, and complex AF substrate management. Over time, a system may paradoxically create more redo cases, more post-ablation tachycardias, and more fragmented care—driving cost and undermining trust.

## 4 Leadless pacemakers: pacing is not deployment—it is physiology

Leadless pacing is frequently portrayed as a technical solution to a technical problem: obtain femoral access, advance the delivery system, secure fixation, confirm thresholds, and release. This procedural simplification narrative is attractive—but incomplete. Pacing is not the act of placing a device. It is the long-term modulation of cardiac electrophysiology. Leadless systems eliminate leads and pockets; they do not eliminate the biological complexity of conduction disease.

### 4.1 Where EP is indispensable

a) Indication nuance and pacing strategy

Avoiding leads and subcutaneous pockets is not, by itself, a sufficient indication. The fundamental question is not “Can this patient receive a leadless pacemaker?” but rather “Is a single-chamber ventricular pacing or apical dual pacing strategy physiologically appropriate for this patient's current and future conduction profile?”

Permanent AF with symptomatic bradycardia is among the most robust indications for single-chamber ventricular pacing leadless pacing. In this context, atrioventricular (AV) synchrony is already absent, and ventricular pacing suffices for rate support. Yet even here, electrophysiologic nuance matters: is the patient truly in permanent AF, should persistent AF be treated with rhythm control, or does paroxysmal sinus rhythm intermittently occur? is there tachy-brady physiology which may be treated with ablation? is AV nodal ablation being contemplated? will conduction system pacing be more appropriate?

Sinus node dysfunction (SND) presents a more complex decision landscape. While some patients with SND and intact AV conduction may benefit from atrium-only pacing, others may derive substantial benefit from dual-chamber support to preserve AV synchrony. Historically, dual-chamber pacing has shown reductions in AF incidence and improved hemodynamics compared with ventricular-only pacing in SND populations [10]. Implanting a ventricular-only leadless system in SND without careful assessment may predispose to: pacemaker syndrome; increased AF burden, and hemodynamic compromise. Emerging AV synchronous leadless technologies mitigate some of these concerns, but atrial mechanical sensing algorithms have known limitations, particularly at higher sinus rates or during atrial arrhythmias [11,12]. Understanding these constraints is not a deployment issue—it is an electrophysiologic one. In patients undergoing AV node ablation, ventricular pacing burden will approach 100%. The long-term impact of chronic right ventricular (RV) pacing on ventricular function must be considered. Evidence from transvenous systems has demonstrated pacing-induced cardiomyopathy in a subset of patients with high RV pacing burden [13]. Although leadless systems avoid leads, they do not eliminate dyssynchrony-related remodeling. Anticipating this risk requires EP-level longitudinal thinking.

Leadless pacing decisions should explicitly address: probability of future dual-chamber need; risk of ventricular dysfunction requiring cardiac resynchronization therapy; potential implantable cardioverter defibrillator indications (e.g., progressive cardiomyopathy); emerging role of conduction system pacing; and management options at battery depletion. Transvenous systems allow relatively straightforward system upgrades. Leadless strategies may require device abandonment, retrieval, or hybrid solutions. While retrieval is feasible early after implantation, chronic retrieval becomes more complex. Implanting multiple intracardiac devices over time raises concerns regarding cumulative device burden, right ventricular occupancy, and valve regurgitation.

b) Device behavior literacy

Understanding rate response, mode switching behavior, atrial mechanical sensing algorithms (where relevant), and the clinical impact of oversensing/undersensing demands EP expertise. A perfectly deployed device can still be clinically wrong if its behavior is misinterpreted or mismanaged [14].

c) Managing complications and extraction pathways

Leadless devices introduce new failure modes: elevated thresholds, dislodgement (rare but real), device-device interactions in multi-device scenarios, and the strategic decision between abandonment vs retrieval when upgrading [15]. EP programs are structurally better positioned to create protocols for these scenarios and to maintain competency in rare-event management.

d) Remote monitoring and follow-up governance

Leadless pacing thrives on monitoring ecosystems. Without a robust EP-led follow-up pipeline, problems surface late, visits are fragmented, and clinical value erodes.

If leadless pacing is treated as “another cath lab implant,” systems may scale volume quickly—but at the cost of inconsistent indication discipline, suboptimal programming, and weak longitudinal outcomes. EP ownership is the safeguard against that drift.

## 5 LAAO: a structural procedure with an EP mandate

LAAO sits at the intersection of structure and rhythm. Some argue it should be “structural IC territory” because it is a closure device in the left atrium. Yet the clinical rationale for LAAO is inseparable from EP's foundational domain: AF management and stroke prevention in rhythm patients.

### 5.1 Why LAAO belongs within EP (with collaboration, not isolation)

a) The indication is an AF decision, not an anatomy decision

LAAO is fundamentally about stroke prevention when anticoagulation is contraindicated or not tolerated, and about balancing ischemic vs bleeding risk in complex AF patients [16]. EP clinicians typically own that decision-making continuum: AF burden interpretation, rhythm strategy, anticoagulation conversations including emerging evidence on whether anticoagulation can be stopped after ablation, and long-term monitoring.

b) LAAO is not “set-and-forget”

Post-implant management is nuanced: antithrombotic regimen tailoring, device-related thrombus surveillance, peri-device leak interpretation, and integration with rhythm interventions. EP programs are structurally oriented toward ongoing arrhythmia care, which is where LAAO patients live.

c) Procedural co-location with AF ablation makes clinical sense

In many systems, AF ablation and LAAO are increasingly co-managed: shared imaging (TEE/ICE), shared left atrial access skills, and shared complication management pathways [17]. Consolidating these within an EP-led rhythm program can reduce fragmentation and streamline patient pathways—provided training and governance are robust.

**Fig. 1:**
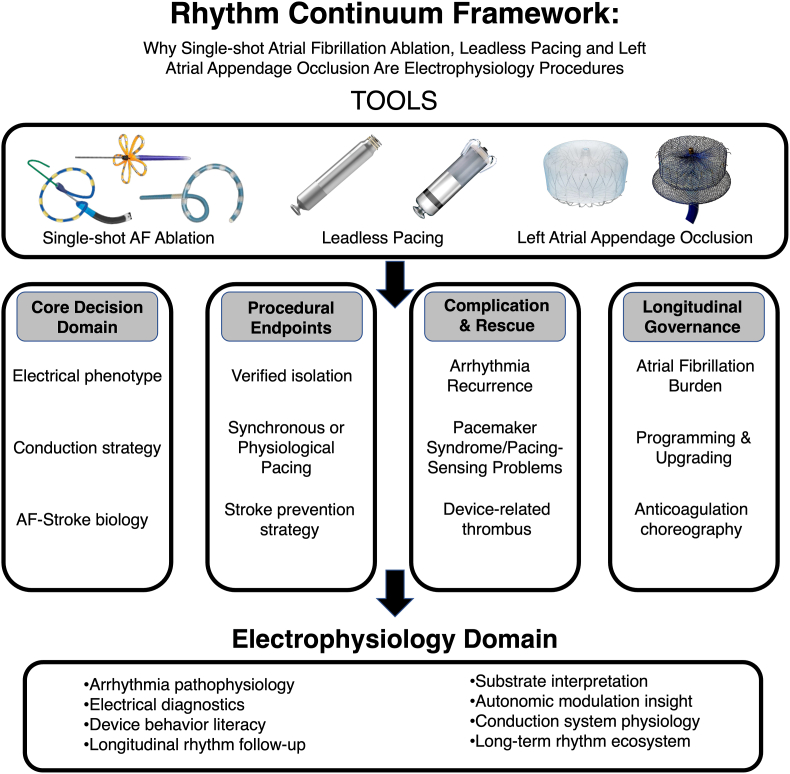

### 5.2 A balanced acknowledgement (Fig. 1)

Yes, LAAO requires structural imaging proficiency and device-specific implantation skill. High-quality LAAO programs often depend on multidisciplinary collaboration (EP, imaging cardiology, anesthesia, IC). A pragmatic model is EP and IC-led clinical ownership with shared procedural credentialing and imaging partnership.

The uncomfortable truth: “procedural drift” is a systems problem, not a people problem.

When non-EP operators adopt EP-adjacent procedures, it is rarely because of individual overreach. It is usually because systems face:

EP workforce shortages.

Long waitlists and access pressure.

Financial incentives aligned to volume.

Marketing-driven “new technology adoption” cycles.

Ambiguous credentialing and governance frameworks.

Therefore, the solution cannot be an emotional argument. It must be an operational blueprint: training standards, credentialing, outcomes reporting, and integrated care pathways.

## 6 A way forward: a practical governance blueprint for the EP community

1) AF ablation, leadless pacing, and LAAO should be done by trained cardiac electrophysiologists. LAAO often requires multidisciplinary collaboration. Competency in these procedures also requires demonstration of satisfactory outcomes.

Credentialing should specify:

Training in electrophysiology.

Case volume thresholds.

Endpoint literacy (PVI verification standards, pacing programming competency, LAAO imaging protocols).

Complication management pathways (tamponade drills, phrenic nerve protocols, device embolization response, DRT management).

Longitudinal follow-up obligations (remote monitoring, rhythm surveillance, anticoagulation strategy, redo pathways, registry participation).

2) Create “Rhythm Programs,” not procedure silos

High-performing centers should organize these therapies under a Rhythm Program with:

Unified patient selection conferences.

Shared imaging workflows (TEE/ICE).

Standard antithrombotic and follow-up protocols.

Outcome dashboards (AF burden, redo rate, complications, DRT, bleeding, stroke/TIA, device performance metrics).

3) Protect the cognitive craft of EP

EP societies should double down on:

Advanced mapping literacy as a core skill.

AF, redo AF and atrial tachycardia ablation expertise as a protected competency.

Device physiology education (not only implant technique).

Autonomic and substrate concepts as the “why” behind the “how”

Otherwise, EP risks being rebranded as “the people who deploy devices”—which is both inaccurate and strategically dangerous.

5) Build transparent outcomes registries

National societies can lead registries capturing:

Procedure type, operator training pathway, center volume.

Complications, including standardized definitions.

Longitudinal outcomes (AF burden metrics, stroke/bleeding outcomes, reinterventions, device performance).

Registries end scope debates quickly because data clarifies where quality is reproducible and where it is not.

In conclusion, AF ablation, leadless pacemakers, and LAAO sit on a continuum of rhythm medicine, with AF catheter ablation performed by cardiac electrophysiologists, leadless pacemakers primarily implanted by electrophysiologists, and LAAO performed with shared decision making and multidisciplinary collaboration. While the procedural tools may appear “simpler,” the clinical decision-making, endpoints, and follow-up are profoundly EP-centric. Reclassifying these as generic interventional procedures risks fragmenting care, diluting standards, and ultimately compromising outcomes. The EP community should respond not with defensiveness, but with governance: clear competency standards, EP-led rhythm programs, structured collaboration with imaging and interventional colleagues, and transparent outcomes reporting. That is the way forward—protecting patients, preserving expertise, and enabling sustainable innovation.

## Declaration of competing interest

The authors declare that they have no known competing financial interests or personal relationships that could have appeared to influence the work reported in this paper.
